# Morning Versus Evening Dosing of Sublingual Immunotherapy in Allergic Asthma: A Prospective Study

**DOI:** 10.3389/fped.2022.892572

**Published:** 2022-06-09

**Authors:** Feng Liao, Shi Chen, Ling Wang, Ying-yu Quan, Li-li Chen, Guo-hua Lin

**Affiliations:** ^1^Center for Prevention and Treatment of Pediatric Asthma, Hainan General Hospital, Hainan Affiliated Hospital of Hainan Medical University, Hainan, China; ^2^Respiratory Department, Geological Hospital of Hainan Province, Hainan, China

**Keywords:** sublingual immunotherapy, allergen-specific immunotherapy, allergic asthma, chronotherapy, house dust mite

## Abstract

**Background:**

Sublingual immunotherapy (SLIT) has been proved to be an effective and safe treatment for allergic asthma (AS) in children. Nonetheless, several issues regarding SLIT remain to be resolved, including the information about optimal administration timing.

**Methods:**

A total of 163 AS children aged 4-13 years were enrolled and randomized into the morning dosing (MD) group and the evening dosing (ED) group. Participants received SLIT with *Dermatophagoides farinae* drops between 7:00 a. m. and 9:00 a.m. (for the MD group) or between 8:00 p. m. and 10:00 p.m. (for the ED group). The total asthma symptom score (TASS), total asthma medicine score (TAMS), Asthma Control Questionnaire (ACQ), forced expiratory volume in one second (FEV_1_), FEV_1_/forced volume vital capacity (FVC), fractional exhaled nitric oxide (FeNO) and adverse events (AEs) were assessed at baseline, 0.5 and 1 year during the 1-year SLIT.

**Results:**

After 1 year, 62 patients in the MD group and 63 patients in the ED group completed the entire study. The clinical efficacy, pulmonary function and FeNO in both groups improved significantly at 0.5 and 1 year (*p* < 0.001). Compared to the MD group, the ED group showed significant lower ACQ score at 0.5 year (*p* < 0.001) and lower FeNO at 1 year (*p* < 0.05). No significant difference between two groups was observed in AE rate (*p* > 0.05). All AEs occurred in the first month, with no systemic AEs reported.

**Conclusion:**

1-year house dust mite (HDM) SLIT is effective and well-tolerated in AS children regardless of administration time. SLIT dosing in the evening might enhance the asthma control level and reduce FeNO level compared with SLIT dosing in the morning.

## Introduction

Sublingual immunotherapy (SLIT) has been proved to be an effective and safe treatment for allergic asthma (AS) in children (1–3). It has disease-modifying properties and confers long-term clinical benefit after cessation of treatment, as demonstrated by prevention of both the onset of new allergic sensitizations and disease progression (4, 5). Nonetheless, several issues regarding SLIT remain to be resolved, including the need of established biomarkers and information about the longer-term effectiveness, cost-effectiveness and optimal administration timing (6, 7).

SLIT is an approach to induce immune tolerance toward specific allergens via sublingual administration of standardized allergen tablets or allergen drops. After intake, antigen-presenting cells (APCs) that distributed in the oral mucosa capture antigens and migrate to regional lymph nodes (8). Dendritic cells (DCs) are the most potent APCs in the mechanism of SLIT that express the high affinity receptor for immunoglobulin E (FcεRI), major histocompatibility complex (MHC) class I and II complexes, and accessory molecules, therefore induce the mucosal tolerance (9, 10). The existing research conjectured that the macrophages/DCs uptake of antigens administered sublingually might be temporally regulated in a circadian manner (11). Other studies suggested the possibility of circadian change in number and phenotypes of sublingual mucosa DCs (12). Due to the key position of DCs in SLIT, it is conjectured that SLIT applied at the certain time could maximize effectiveness and minimize side effects, which still needs further research (13, 14). In this study, we compared the efficacy and safety of SLIT performed at two different time points (in the morning and in the evening) in AS children and sought to identify when to receive SLIT could be more beneficial.

## Materials and Methods

### Study Design

Subjects aged 4-13 years were recruited from outpatients that visited the Hainan General Hospital from August 2019 to April 2021. Eligible participants were randomly assigned to the morning dosing (MD) group and the evening dosing (ED) group by a computer-generated randomization method and stratified by sensitization status (monosensitized vs. polysensitized). All participants received daily SLIT between 7:00 a. m. and 9:00 a.m. (for the MD group) or between 8:00 p. m. and 10:00 p.m. (for the ED group) with glycerinated *Dermatophagoides farinae* drops. The standardized drops (Chanllergen; Zhejiang Wolwo Bio-Pharmaceutical Co., Ltd., Zhejiang, China) were labeled from 1 to 4 with the total protein concentration of 1, 10, 100, and 333 μg/mL, respectively. The drops were self-administered sublingually for 1-3 min before swallowing. Nothing was allowed by mouth for 15 min after the administration of the drops.

All patients were required to take daily doses in strict accordance with the manufacturer’s instructions. The young children were administered the SLIT extracts under the supervision of their guardians. In the first 3 weeks, participants were instructed to take drops No. 1, drops No. 2 and drops No. 3, respectively, as increment phase, in a gradually increasing order of 0.05, 0.10, 0.15, 0.20, 0.30, 0.40, 0.50 mL/day. From week 4, patients were treated with 0.15 mL of drops No. 4/day until the treatment completed (15, 16).

Investigators collected participants’ demographic characteristics and clinical data before initiation of treatment. Telephone follow-ups were provided to patients monthly to supervise medication and assess adverse events (AEs). Patients returned to hospital at 0.5 and 1 year for evaluation, including clinical efficacy, lung function and the FeNO level. The efficacy and safety analysis only involved data of patients who completed the study, while the data of patients who did not complete the study were excluded. The present study was approved by the Ethics Committee of Hainan General Hospital and conducted in compliance with the Ethical Guidelines for Clinical Studies and Good Clinical Practice. All patients and their guardians were informed of the relevant information prior to their participation in the study (17).

### Participants

Patients with AS in remission due to house dust mite (HDM) were enrolled into the study. Inclusion criteria were as follows: all patients were diagnosed with mild-to-moderate AS according to Global Initiative for Asthma (GINA) (18); patients were sensitized to *D. farinae* and/or *D. pteronyssinus*. Mild asthma is defined as asthma that is well controlled with as-needed ICS-formoterol alone. or with low-intensity maintenance controller treatment such as low dose ICS, leukotriene receptor antagonists or chromones. Moderate asthma is defined as asthma that is well controlled with low or medium dose ICS-LABA. Patients only sensitized to *D. farinae* and/or *D. pteronyssinus* were allocated to the monosensitized group, while patients who were sensitive to *D. farinae* and/or *D. pteronyssinus* and other coexisting inhaled allergens were allocated to the polysensitized group. Sensitization to *D. farinae*, *D. pteronyssinus* and other aeroallergens were further confirmed by the presence of specific immunoglobulin E (sIgE) ≥ 0.7 KU/L, using UniCAP system (Phadia, Uppsala, Sweden). Exclusion criteria were as follows: forced expiratory volume in one second (FEV_1_) < 70% of predictive value; severe systemic diseases such as poorly controlled cardiovascular diseases, immune diseases, or malignant tumors; receiving β-blockers or angiotensin-converting enzyme inhibitors; serious psychological barriers or failed to understand the risks and limitations of treatment.

### Clinical Efficacy

Participants or their guardians were required to record symptom and medication consumption 10 min before the SLIT administration daily throughout the study period. The investigators calculated the weekly average scores at every visit. The total asthma symptom score (TASS) was the sum of daytime asthma symptom scores and nighttime asthma symptom scores (1). The daytime asthma symptoms were scored based on a scale of 0-5 points, in accordance with the general severity of asthma symptoms (wheeze, shortness of breath, dyspnea, and cough) and its impact on daily life. 0, no symptoms; 1, symptoms are rare and short lasting; 2, two or more short lasting symptoms; 3, mild symptoms for more of the day, but had little impact on life and work; 4, severe symptoms for more of the day and affect life and work; 5, the symptom is so serious that the subject cannot work and live normally. The nighttime asthma symptoms were scored based on a scale of 0-4 points, in accordance with the frequency of nocturnal and early morning awakening induced by asthma. 0, no symptoms; 1, wake up once or wake up early; 2, wake up twice, including wake up early; 3, wake up many times (≥ 3 times); 4, can’ t fall asleep at night. Patients were prescribed pharmacologic therapy for symptom remission and required to use according to physicians’ instruction. Rescue medication score was calculated as follows (per day): 0 = no use of relief medication; 1 = use of oral antihistamines, anti-leukotrienes, or β2 receptor agonists; 2 = use of inhaled corticosteroids; 3 = use of combination therapy (corticosteroids with β2 receptor agonists). The total asthma medicine score (TAMS) was the sum of all the recorded medicine scores (19). The Asthma Control Questionnaire (ACQ) has strong evaluative and discriminative properties and can be used with confidence to measure asthma control. The ACQ includes five questions about the most important symptoms for asthma control assessment, one question about β2 agonist use and another about FEV_1_. The items are equally weighted and the ACQ score is the mean of the 7 items and therefore between 0 (well controlled) and 6 (extremely poorly controlled) (20).

### Pulmonary Function

The pulmonary function of the children was determined by the Master Screen lung function instrument (Jaeger GmbH, Cologne, Germany) at every visit. The measurement parameters included FEV_1_ and FEV_1_/forced volume vital capacity (FVC) (21).

### Fractional Exhaled Nitric Oxide

The fractional exhaled nitric oxide (FeNO) was evaluated at every visit by using the exhaled nitric oxide tester (SUNVOU, Wuxi, China). FeNO level was detected in accordance with the FeNO standardized monitoring guidelines recommended by the American Thoracic Society (ATS) and the European Respiratory Society (ERS) (22). The children seated comfortably and breathed quietly for approximately 5 min, then inhaled the gas contain low NO concentration (< 5 ppb) to near total lung capacity (TLC) and immediately exhaled at a constant flow rate of 50 ml/second, until an NO plateau of at least 2 s could be identified during an exhalation of at least 4 seconds.

### Adverse Events

The occurrence rate, duration, and severity (23) were recorded to assess AEs. All AEs were addressed under the instruction of the physicians.

### Statistical Analysis

Statistical analysis was performed in demographic analysis and efficacy assessment with IBM SPSS Statistics 21.0. The differences between the MD group and the ED group at baseline demographic characteristics were analyzed by χ^2^ test. The intragroup comparisons of clinical characteristics were performed by the Friedman M test. The intergroup comparisons of clinical characteristics were performed by the Mann-Whitney U test. The 2-tailed level of statistical significance was set at *p* = 0.05.

## Results

### Study Participants

**FIGURE 1:**
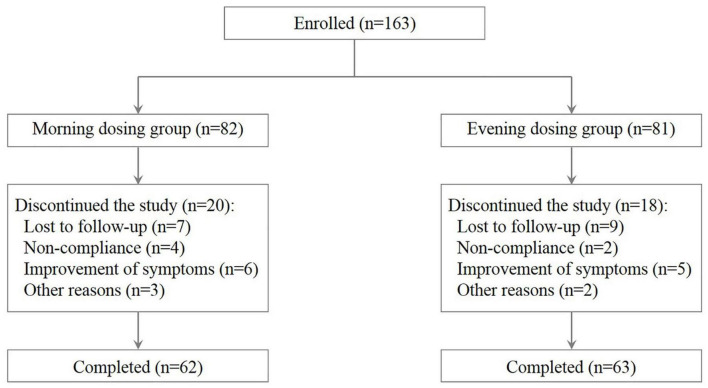
Flow diagram of participant disposition.

A total of 163 patients [mean age 8.39 ± 2.69 years, 24.54% female (*n* = 40), 75.46% male (*n* = 123)] fulfilling the eligibility requirements were randomized and divided into two groups (Figure 1). The MD group and the ED group included 82 patients and 81 patients, respectively. After 1 year, 62 patients in the MD group (75.61%) and 63 patients in the ED group (77.77%) completed the entire study. Reasons for discontinuation were as follows: lost to follow-up (n = 16), protocol non-compliance (n = 6), improvement of symptoms (n = 11) and other reasons (n = 5). Both groups were well balanced in respect of age, gender and sensitization status (Table 1, all *p* > 0.05). Similarly, there was no statistical difference in all items of clinical data between two groups (all *p* > 0.05).

**TABLE 1:** Characteristics of the study subjects. Data are presented as n (%) or Mean ± SD as appropriate.

Characteristics	MD group (*n* = 62)	ED group (*n* = 63)	*p*-value
**Age**			
Mean, years	7.92 ± 2.52	8.70 ± 2.27	> 0.05
**Gender**			
Male	44 (70.97%)	48 (76.19%)	> 0.05
Female	18 (29.03%)	15 (23.81%)	> 0.05
**Sensitization status**			
Monosensitized	4 (6.45%)	2 (3.17%)	> 0.05
Polysensitized	58 (93.55%)	61 (96.83%)	> 0.05
**Clinical data**			
Baseline TASS	3.03 ± 0.79	3.19 ± 0.91	> 0.05
Baseline TAMS	5.03 ± 0.18	5.00 ± 0.00	> 0.05
Baseline ACQ	1.66 ± 0.33	1.53 ± 0.44	> 0.05
Baseline FeNO (ppb)	29.47 ± 14.01	26.08 ± 14.04	> 0.05
Baseline FEV_1_ (%)	86.03 ± 10.05	83.63 ± 7.99	> 0.05
Baseline FEV_1_/FVC (%)	91.16 ± 9.07	91.11 ± 7.29	> 0.05

*p-values designate significant difference between the MD group and the ED group.*

### Clinical Efficacy

**FIGURE 2:**
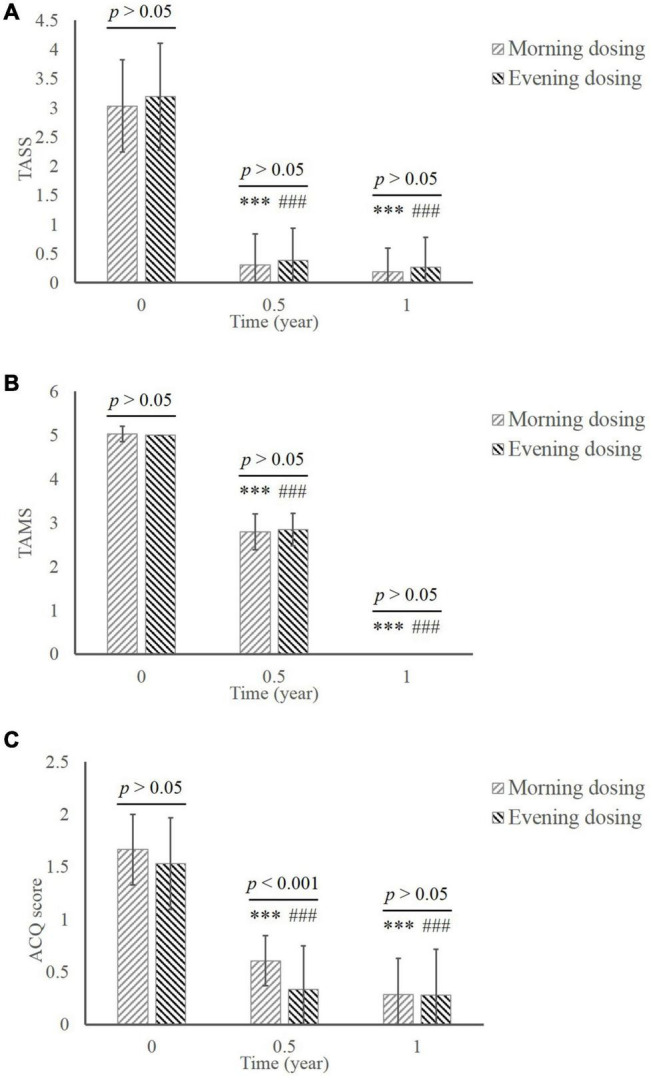
The comparison of clinical efficacy between the MD group and the ED group. **(A)** TASS. **(B)** TAMS. **(C)** ACQ. ^***^, *p* < 0.001, significant difference in the MD group compared to baseline; ^###^, *p* < 0.001, significant difference in the ED group compared to baseline. MD, morning dosing; ED, evening dosing; TASS, total asthma symptom score; TAMS, total asthma medicine score; ACQ, Asthma Control Questionnaire.

The TASS of patients in both groups decreased significantly during the treatment in comparison of baseline (Figure 2A; MD group, 3.03 ± 0.79 at baseline vs. 0.31 ± 0.53 in 0.5 year vs. 0.19 ± 0.40 in 1 year; ED group, 3.19 ± 0.91 at baseline vs. 0.38 ± 0.55 in 0.5 year vs. 0.27 ± 0.51 in 1 year; all *p* < 0.001compared to baseline). The significant reduction of TAMS was consistent with the TASS results (Figure 2B; MD group, 5.03 ± 0.18 at baseline vs. 2.79 ± 0.41 in 0.5 year vs. 0.00 ± 0.00 in 1 year; ED group, 5.00 ± 0.00 at baseline vs. 2.84 ± 0.37 in 0.5 year vs. 0.00 ± 0.00 in 1 year; all *p* < 0.001 compared to baseline). No significant difference between MD and ED group was shown in those clinical scores (*p* > 0.05).

The score of ACQ declined from baseline to the end of treatment in both groups (Figure 2C; MD group, 1.66 ± 0.33 at baseline vs. 0.61 ± 0.24 in 0.5 year vs. 0.29 ± 0.34 in 1 year; ED group, 1.53 ± 0.44 at baseline vs. 0.33 ± 0.42 in 0.5 year vs. 0.28 ± 0.44 in 1 year; all *p* < 0.001 compared to baseline). Notably, significantly greater improvement in ACQ was manifested in the ED group compared with the MD group at 0.5 year (*p* < 0.001). This difference disappeared by the end of the study.

### Pulmonary Function

**FIGURE 3:**
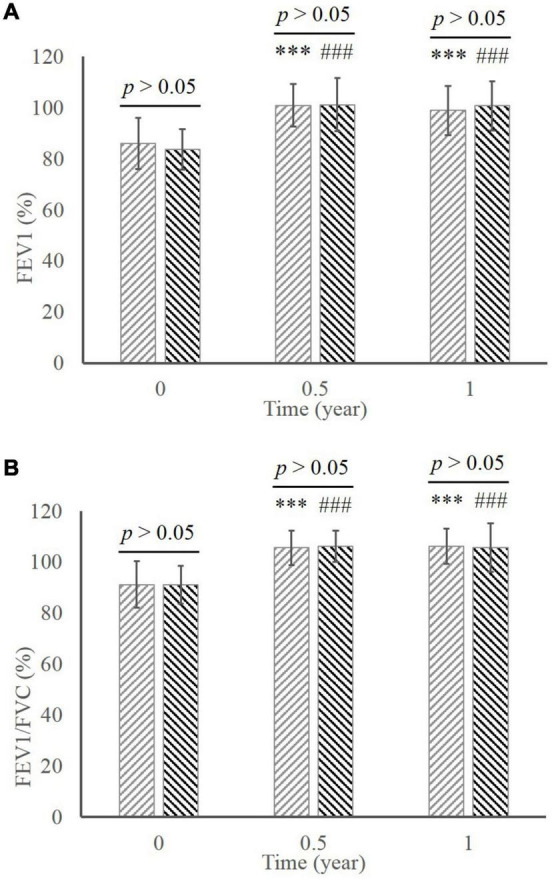
The comparison of pulmonary function between the MD group and the ED group. **(A)** FEV_1_. **(B)** FEV_1_/FVC. ^***^, *p* < 0.001, significant difference in the MD group compared to baseline; ^###^, *p* < 0.001, significant difference in the ED group compared to baseline. MD, morning dosing; ED, evening dosing; FEV_1_, forced expiratory volume in one second; FEV_1_/FVC, FEV_1_/forced volume vital capacity.

There were significant increases in FEV_1_ (Figure 3A; MD group, 86.03 ± 10.05 at baseline vs. 100.89 ± 8.27 in 0.5 year vs. 98.92 ± 9.66 in 1 year; ED group, 83.63 ± 7.99 at baseline vs. 101.17 ± 10.29 in 0.5 year vs. 100.70 ± 9.57 in 1 year; all *p* < 0.001 compared to baseline) and FEV_1_/EVC (Figure 3B; MD group, 91.16 ± 9.07 at baseline vs. 105.60 ± 6.73 in 0.5 year vs. 106.19 ± 6.99 in 1 year; ED group, 91.11 ± 7.29 at baseline vs. 106.16 ± 6.19 in 0.5 year vs. 105.68 ± 9.44 in 1 year; all *p* < 0.001 compared to baseline) during the whole treatment in both groups. Comparison in respect of pulmonary function revealed no significant difference between two groups throughout the treatment (*p* > 0.05).

### Fractional Exhaled Nitric Oxide

**FIGURE 4:**
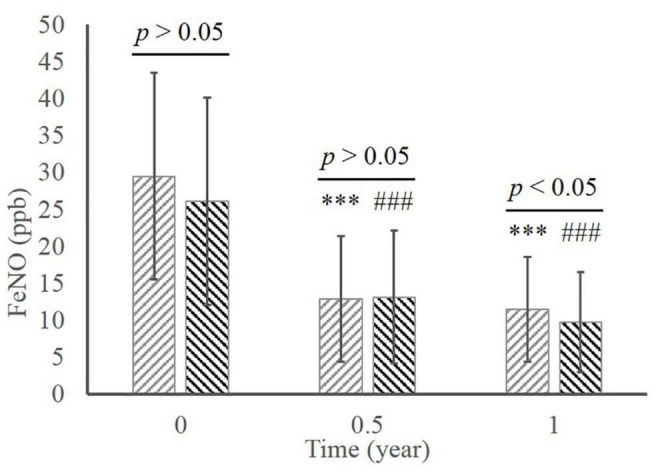
The comparison of FeNO between the MD group and the ED group.^ ***^, *p* < 0.001, significant difference in the MD group compared to baseline; ^###^, *p* < 0.001, significant difference in the ED group compared to baseline. MD, morning dosing; ED, evening dosing; FeNO, fractional exhaled nitric oxide.

In the MD group, there was significant decrease of FeNO level during the whole therapy (Figure 4; 29.47 ± 14.01 at baseline vs. 12.84 ± 8.48 in 0.5 year vs. 11.42 ± 7.12 in 1 year, all *p* < 0.001 compared to baseline). The level of FeNO also significantly ameliorated in the ED group (26.08 ± 14.04 at baseline vs. 13.10 ± 8.98 in 0.5 year vs. 9.75 ± 6.78 in 1 year, all *p* < 0.001 compared to baseline), whereas a significant better improvement than the MD group occurred at 1 year (*p* < 0.05).

### Safety

No deaths, anaphylactic shocks or life-threatening events were reported during the entire study (Table 2). Seven AEs that occurred in the MD group were 3 oral numbness or itching, 3 local rash, and 1 asthma attack. Three AEs that occurred in the ED group were 1 oral numbness or itching, and 2 fatigue. The AE rate was 11.29 and 4.76% in the MD group and the ED group, respectively, with no statistically significant difference between the two groups (*p* > 0.05). All AEs occurred in the first month of SLIT, and mitigated with or without medication treatment within a week.

**TABLE 2:** Reported AEs in the study.

	MD group (*n* = 62)	ED group (*n* = 63)
Total no. of AEs	7	3
Oral numbness or itching	3	1
Local rash	3	0
Asthma attack	1	0
Fatigue	0	2

## Discussion

Asthma is a major public health burden affecting around 350 million people worldwide (24). In China, a cross-sectional survey involving approximately 0.5 million children aged 0-14 years indicated a growing prevalence of AS from 1.09% in 1990 to 3.02% in 2010 (25). SLIT has been used globally for more than 30 years. Its efficacy and safety for AS have been confirmed from several trials (5, 26–28). In our study, we observed that after 1 year of treatment, TASS, TAMS, ACQ score and FeNO decreased, whereas the FEV_1_ and FEV_1_/FVC increased, without any severe systematic AEs occurred. These results demonstrated that SLIT can achieve improvement in aspect of clinical efficacy, pulmonary function and the FeNO level among pediatric patients with HDM-driven AS.

The GINA report recommended the assessment of asthma control in two domains: symptom control and risk factors for future poor outcomes. Lung function is an important part of the assessment of future risk and should be measured periodically throughout the treatment (18). Heretofore, there is still a lack of further research on the effect of SLIT in lung function (29, 30). A retrospective analysis reviewed 31 cases of AS patients with/without allergic rhinitis (AR), revealed that the FEV_1_ of AS children improved significantly in 1 year of SLIT (31). In addition to the amelioration in asthma symptoms and medication use, we also found significant pulmonary function improvement of AS patients in this study, i.e., an ascending level of FEV_1_ and FEV_1_/FVC. These results were consistent with the previous studies (31, 32).

The nitric oxide (NO) has been playing an important role in the upper/lower airway inflammation as an inflammatory mediator. Recently, Parisi GF and colleagues observed a statistically significant reduction of nasal nitric oxide (nNO) after 6 months of SLIT, suggested that nNO could be one of the predictive biomarkers of short-term efficacy of SLIT in HDM-induced AR patients (33). FeNO is higher in asthma that is characterized by type 2 airway inflammation (34). As a typical type 2 airway inflammation-mediated disease, AS is therefore often considered to be associated with elevated FeNO (35). However, there is limited evidence for the effects of SLIT on FeNO in AS (36, 37). Wang et al. reported a significant reduction of FeNO in AS children undergoing SLIT (38). The mentioned study enrolled 200 asthma children and divided into two groups. After 1 year, the SLIT group exhibited lower FeNO than before treatment, and the decreased value of FeNO was higher than that in the control group. In the present study, the FeNO of AS patients continued to decline in 1-year treatment. It was speculated that HDM SLIT could alleviate airway inflammation.

Several medicines have been reported to exhibit administration-time-related-effects in allergic diseases because of the circadian rhythm of symptoms (39, 40). Nevertheless, most findings focus on pharmacologic therapy instead of allergen immunotherapy (41–43). Studies of SLIT have proposed several mechanisms, including the regulation of T cell responses and the inhibition of impaired antibody production (10). Igarashi and colleagues found a greater decrease in total and allergen-specific IgE levels in the resting phase, which was correlated with a reduction of allergen-specific T cell responses, revealed that SLIT in mice may be more effective in the resting phase than that in the active phase (11). Possible reasons for these results included the circadian changes in antigen uptake by macrophages/DCs and circadian changes in sublingual mucosa DCs. Thus, we wondered whether the use of chronotherapeutic approach for SLIT in AS children might differ the efficacy. Ultimately, we did not observe significant difference in TASS, TAMS, FEV_1_ and FEV_1_/FVC between the MD group and the ED group, thereby suggesting that regardless of dosing regimen, SLIT in pediatric patients with HDM-driven AS could improve asthma symptoms, medication use and lung function effectively. Interestingly, we noticed that the ACQ score of the ED group was lower than the MD group in 0.5 year, while the FeNO of the ED group was lower than the MD group in 1 year. These findings lead to the conclusion that SLIT performed during the nighttime might be more beneficial in asthma control and the FeNO level than that of daytime.

The safety of SLIT has been demonstrated in an abundance of published data (44–46). In this study, no serious AEs were reported in all patients. All AEs were mild, and relieved with or without medication treatment within a week. These results were consistent with previous reports (47). Meanwhile, there was no significant difference in the AE rate between the MD group and the ED group, confirming similar safety of morning dosing SLIT and evening dosing SLIT.

The main limitation in this study is the lack of double-blind design and there is absence of a placebo drop. Since the majority of patients we recruited were polysensitized, it is difficult to explain whether the conclusions of our study are different in monosensitized subgroup or polysensitized subgroup. We are preparing a double-blinded, placebo-controlled study with a larger sample size to further confirm the conclusions of this study. And investigate the influence of HDM environmental concentration and sensitization status.

In conclusion, 1-year HDM SLIT provided efficacy and safety in pediatric patients with AS irrespective of dosing schedule. SLIT administrated in the evening might be more beneficial in asthma control and the FeNO level than SLIT administrated in the morning.

## Data Availability Statement

The raw data supporting the conclusions of this article will be made available by the authors, without undue reservation.

## Ethics Statement

The studies involving human participants were reviewed and approved by the Ethics Committee of Hainan General Hospital. Written informed consent to participate in this study was provided by the participants’ legal guardian/next of kin.

## Author Contributions

FL and SC designed the study and wrote the manuscript. LW and G-HL examined the patients and collected the data. Y-YQ and L-LC analyzed the data and performed the statistical analysis. SC and G-HL supervised the study and critically reviewed the manuscript. All authors contributed to the article, significantly to the study, and approved the submitted version.

## Conflict of Interest

The authors declare that the research was conducted in the absence of any commercial or financial relationships that could be construed as a potential conflict of interest.

## Publisher’s Note

All claims expressed in this article are solely those of the authors and do not necessarily represent those of their affiliated organizations, or those of the publisher, the editors and the reviewers. Any product that may be evaluated in this article, or claim that may be made by its manufacturer, is not guaranteed or endorsed by the publisher.
